# Identification and Evolution of Drug Efflux Pump in Clinical *Enterobacter aerogenes* Strains Isolated in 1995 and 2003

**DOI:** 10.1371/journal.pone.0003203

**Published:** 2008-09-12

**Authors:** Jacqueline Chevalier, Céline Mulfinger, Eric Garnotel, Pierre Nicolas, Anne Davin-Régli, Jean-Marie Pagès

**Affiliations:** 1 UMR-MD1, IFR 48, Facultés de Médecine et de Pharmacie, Marseille, France; 2 Service de Biologie, H.I.A. Laveran, Marseille, France; 3 IMTSSA, Marseille, France; The Scripps Research Institute, United States of America

## Abstract

**Background:**

The high mortality impact of infectious diseases will increase due to accelerated evolution of antibiotic resistance in important human pathogens. Development of antibiotic resistance is a evolutionary process inducing the erosion of the effectiveness of our arsenal of antibiotics. Resistance is not necessarily limited to a single class of antibacterial agents but may affect many unrelated compounds; this is termed ‘multidrug resistance’ (MDR). The major mechanism of MDR is the active expulsion of drugs by bacterial pumps; the treatment of Gram negative bacterial infections is compromised due to resistance mechanisms including the expression of efflux pumps that actively expel various usual antibiotics (ß-lactams, quinolones, …).

**Methodology/Principal Findings:**

*Enterobacter aerogenes* has emerged among *Enterobacteriaceae* associated hospital infections during the last twenty years due to its faculty of adaptation to antibiotic stresses. Clinical isolates of *E. aerogenes* belonging to two strain collections isolated in 1995 and 2003 respectively, were screened to assess the involvement of efflux pumps in antibiotic resistance. Drug susceptibility assays were performed on all bacterial isolates and an efflux pump inhibitor (PAßN) previously characterized allowed to decipher the role of efflux in the resistance. Accumulation of labelled chloramphenicol was monitored in the presence of an energy poison to determine the involvement of active efflux on the antibiotic intracellular concentrations. The presence of the PAßN-susceptible efflux system was also identified in resistant *E. aerogenes* strains.

**Conclusions/Significance:**

For the first time a noticeable increase in clinical isolates containing an efflux mechanism susceptible to pump inhibitor is report within an 8 year period. After the emergence of extended spectrum ß-lactamases in *E. aerogenes* and the recent characterisation of porin mutations in clinical isolates, this study describing an increase in inhibitor-susceptible efflux throws light on a new step in the evolution of mechanism in *E. aerogenes*.

## Introduction

Among *Enterobacteriaceae* associated hospital infections, *Enterobacter aerogenes* has emerged during the last twenty years due to its efficient adaptative response to environmental stresses [1]. This common hospital pathogen in Europe is involved in nosocomial respiratory tract and urinary infectious diseases [2]–[8]. This behaviour has been associated with a decreased susceptibility to the most recently developed cephalosporins, including cefepime and cefpirome (last cephalosporin generation), and to carbapenems [4], [9]–[11]. An increasing number of clinical *E. aerogenes* strains exhibits a plasmid encoding extended-spectrum ß-lactamase associated with an aminoglycoside enzymatic resistance in addition to a chromosomal cephalosporinase and the clinical isolates present also an acquired resistance, *via* target mutation, to other antibiotic classes such as quinolones [2], [4]–[15]. The regulation of envelope permeability including the synthesis of porins, the modification of lipopolysaccharide and the expression of efflux pumps has been reported by several studies:(i) a strong correlation has been reported between the absence of the *E. aerogenes* major porin, Omp36, and imipenem resistance, (ii) some isolates showing a resistance towards polymyxin group had a simultaneous alteration of the lipopolysaccharide structure and, (iii) in multi-drug resistant (MDR) strains the expression of an efflux pump has been demonstrated to contribute to a severe decrease in intracellular concentrations of various antibiotic classes [3], [4], [10], [12], [13], [16]. It has been demonstrated that during 5 days of imipenem treatment, clinical isolates had developed imipenem resistance *via* a decrease of porin synthesis conjointly to a production of efflux system [4], [17]. This tremendous capacity to rapidly develop antibiotic resistance has been associated to the regulation cascade involving the *mar* regulon and *ramA* regulator gene which control the expression of membrane transporters [1]. Bacterial efflux pumps represent an active protection mechanism against toxic compounds and the major efflux pump, AcrAB-TolC, identified in *E. aerogenes* clinical isolates expels a variety of compounds including detergents and structurally unrelated antimicrobial agents such as quinolones, tetracyclines and chloramphenicol [18]–[21]. The activity of efflux pump in various resistant clinical isolates has been characterized by using different efflux pump inhibitors that compete with the antibiotic efflux or with an energy poison that collapses the membrane potential required for the active antibiotic transport [22]–[24].

The poly-specificity of efflux transporters confers a general resistance phenotype that can drive the acquisition of additional mechanisms of antibiotic resistance such as target mutation or secretion of enzymes that degrade antibiotics and also reinforce the effect of these acquired mechanisms [20]. It has been recently demonstrated that the expression of the AcrAB-TolC pump is an important prerequisite for the selection of fluoroquinolone resistant mutants that exhibit mutated targets (DNA gyrase) in various Gram-negative bacteria such as *Salmonella* or *Campylobacter*, two major food-borne pathogens involved in severe human diseases [25], [26]. Consequently, the spread of this resistance mechanism and the modulation of the different bacterial responses to antibiotic therapy calls for the identification of efflux pump activity and their prevalence in clinical strains. An important question addresses the evolution of drug resistance and the dissemination of this efflux mechanism in Gram-negative bacterial pathogens.

To this aim, we decided to analyse two *E. aerogenes* populations isolated from Marseille hospitals in 1995 and 2003. The local criteria used in 1995 [10], were re-used in 2003 in order to prevent (i) introduction of some bias caused by different procedures used by other institutions and (ii) ecological variance due to a plethora of variable associated with different geographic areas. These isolates were selected according to their noticeable cross-resistance pattern to cephalosporins as previously reported [10]. In addition, the epidemiology of the *E. aerogenes* strains are checked in the two collections. We thus compare the two collections for resistance to structurally unrelated antibiotics to assess the frequency of strains exhibiting a MDR phenotype that may reflect the production of an efflux mechanism and to evaluate the number of strains expressing a documented efflux pump during this 1995–2003 interval.

## Results

### Strain characterization and antibiotic susceptibility of *E. aerogenes* isolates

**Figure 1:**
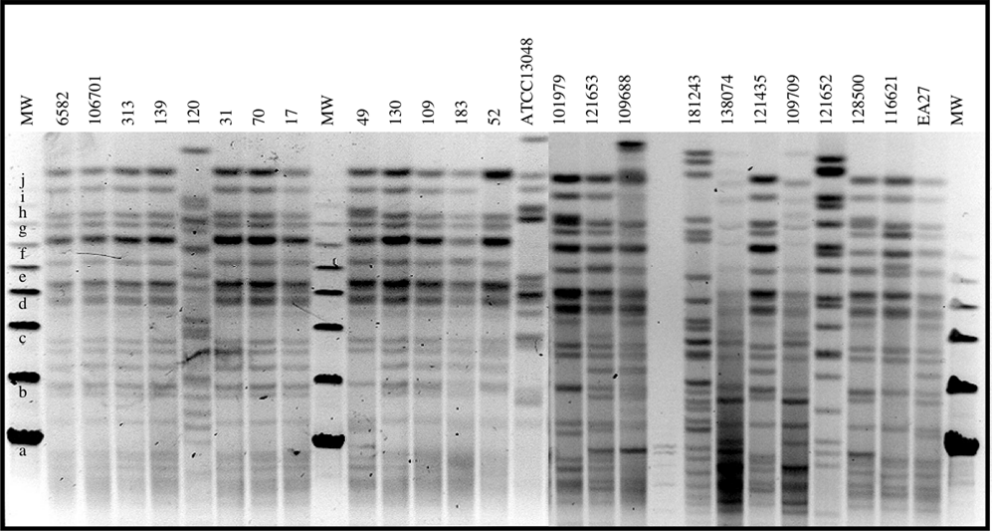
PFGE of *Xba*I-digested genomic DNA preparations from various clinical *Enterobacter aerogenes* isolates. 1995 isolates: 6582, 106701, 313, 139, 120, 31, 70, 17, 49, 130, 109, 183, 52. 2003 isolates: 101979, 121653, 109688, 181243, 138074, 121435, 109709, 121652, 128500, 116621. ATCC13048 and EA27, used as reference strains. Molecular weight markers are indicated: a, 48.5 kb; b, 97 kb; c, 145.5 kb; d, 194 kb; e, 242.5 kb; f, 291 kb; g, 340 kb; h, 388 kb; i, 437 kb; j, 485 kb; h, 534 kb.

The DNA restriction pattern of isolates collected during the years 1995 and 2003 were performed and analysed by pulsed field gel electrophoresis (PFGE) to determine their relatedness. These DNA restriction patterns were compared according to criteria proposed by Tenover *et al.* [27]. Figure 1 illustrates a representative set of this molecular epidemiological typing. Isolates whose patterns were indistinguishable or differing by no more than 3 fragments were considered to belong to the same epidemic clone. An isolate was considered to be possibly related to the outbreak strain if their patterns did not differ in more than 4–6 bands. The patterns were unrelated if they showed more than 7 band differences. Forty-eight strains were studied and 46 generated reproducible fingerprint patterns. Among them the pattern of 21 strains was identical, 14 showed 1 to 5 band differences and 11 had a pattern with more than 6 band differences. Among these eleven strains, 3 isolates were closely related. 35 isolates were considered to belong to the same epidemiological prevalent clone previously reported [4], [5], [9], [28] and 11 strains were consistent to be of different origin.

The antibiotic susceptibilities of strains collected during the periods 1995 and 2003 were presented in Table 1–2. A large number of the isolates exhibited a noticeable resistance phenotype, *e.g.* 61% and 74 to 83% of strains were resistant to chloramphenicol and tested quinolones, respectively (Table 3). Conversely, only 2% and 9% of strains were resistant to imipenem and cefepime respectively. About 12% of tested strains harboured a susceptible phenotype against the used antibiotics (imipenem, cefepime, tetracycline, chloramphenicol, nalidixic acid, norfloxacin, ofloxacin, sparfloxacin). Although chloramphenicol is now rarely used in human therapy, a major proportion of the screened isolates exhibited chloramphenicol resistance. In addition, these strains also presented a high level resistance against other assayed antibiotics.

**Table 1:** Susceptibilities of the *E. aerogenes* isolates (1995) to different antibiotics (MIC µg/mL)^a^.

Strain	IMI	CEF	TET	CHL	NAL	NFL	OFL	SPA
0	1	4	1	256	>512	128	128	64
1	4	1	4	256	>512	256	64	4
5	2	4	1	8	>512	64	32	8
7	0,05	8	16	8	2	0,5	<4	0,06
11	1	8	4	256	>512	128	128	128
12	1	4	1	8	64	0,125	<4	0,5
16	4	128	2	128	32	16	<4	<0,5
17	2	4	16	>256	>512	128	32	32
19	>4	16	8	256	>512	512	128	256
20	4	1	2	128	>512	256	128	64
31	1	16	2	256	>512	128	64	128
34	>4	128	8	256	>512	512	128	128
36	2	4	>256	16	>512	512	256	128
44	4	4	16	>256	>512	256	128	256
49	>4	8	8	>256	>512	512	128	256
52	>4	1	2	64	>512	256	64	16
53	>4	1	8	>256	>512	256	128	256
54	2	64	2	16	>512	64	64	16
56	>4	16	<0,5	256	>512	256	128	128
59	2	1	4	<4	<4	<4	<4	<0,5
70	>4	8	4	>256	512	256	128	256
71	2	4	2	<4	>512	<4	<4	8
76	2	8	1	16	>512	64	64	16
98	1	32	>512	8	128	1	<4	0,5
104	2	32	128	256	>512	64	64	32
108	2	1	2	<4	>512	64	32	8
109	4	4	16	>256	>512	512	128	512
115	0,5	1	2	2	>512	128	16	64
120	1	1	1	256	>512	128	16	64
124	4	1	2	2	4	0,25	<4	0,125
130	4	1	8	>256	>512	128	128	256
135	1	1	1	2	4	0,25	<4	<0,03
138	4	2	4	4	4	<4	<4	<0,5
139	2	32	1	256	>512	64	128	64
148	1	1	2	<4	64	8	<4	<0,5
151	1	4	4	4	8	4	<4	0,06
179	0,5	4	2	256	>512	128	64	128
183	4	128	4	>256	>512	256	64	256
313	2	1	2	128	>512	128	64	128
317	>4	1	64	32	>512	32	32	16
441	4	4	8	>256	>512	256	128	256
701	>4	64	8	>256	>512	512	128	256
6582	4	4	4	256	>512	256	128	256
7237	4	32	4	256	>512	256	128	256
106701	2	1	8	>256	>512	256	128	512

a The values are the means of three independent assays.

Antimicrobial agent abbreviations: IMI, Imipenem; CEF, Cefepime; TET, Tetracycline; CHL, Chloramphenicol; NAL, Nalidixic acid; NFL, Norfloxacin; OFL, Ofloxacin; SPA, Sparfloxacin.

**Table 2:** Susceptibilities of the *E. aerogenes* isolates (2003) to different antibiotics (MIC µg/mL)^a^.

Strain	IMI	CEF	TET	CHL	NAL	NFL	OFL	SPA
100128	1	2	1	8	>512	64	128	64
101234	2	1	4	>256	>512	128	64	64
101451	1	1	2	4	8	0,25	<4	0,06
101979	4	2	4	16	>512	128	128	256
103280	4	1	8	8	16	<4	<4	<0,5
105784	4	4	4	32	>512	256	128	256
105891	2	1	0,5	8	4	0,25	<4	0,06
106206	4	64	4	256	>512	128	128	256
107868	2	8	0,5	128	>512	128	128	128
108055	4	1	16	16	512	8	<4	<0,5
108418	1	2	8	>256	>512	256	128	256
108969	4	1	4	16	16	<4	<4	<0,5
109688	1	4	2	16	>512	128	64	64
109709	4	2	4	16	>512	512	128	256
110199	2	1	0,5	4	8	0,25	<4	0,06
110360	2	1	32	>256	>512	256	128	256
110721	4	1	4	16	>512	512	256	512
111631	2	1	16	>256	>512	256	128	256
112144	4	1	4	16	16	<4	<4	<0,5
112446	4	1	32	32	128	<4	<4	<0,5
112978	4	4	2	<4	>512	256	64	128
113022	2	4	2	256	>512	256	128	64
115264	4	4	8	>256	>512	256	128	256
118259	1	2	4	256	>512	256	128	256
121435	2	8	8	>256	>512	512	128	256
121652	1	1	2	0,5	8	0,25	<4	4
121653	1	1	2	256	>512	256	256	128
121681	2	4	4	256	>512	512	128	256
122554	0,5	4	2	128	>512	64	64	64
122791	2	4	2	64	>512	256	256	128
123258	4	1	8	16	16	<4	<4	<0,5
123369	4	1	8	256	>512	256	128	256
123800	2	1	4	4	8	<4	<4	<0,5
128500	1	2	4	256	>512	128	128	128
128553	>4	8	8	>256	>512	128	128	256
129689	4	4	8	>256	>512	256	64	256
131102	4	2	8	>256	>512	256	128	256
131150	0,5	2	4	256	>512	128	128	128
131538	>4	32	8	>256	>512	256	128	256
134146	2	1	4	32	>512	128	64	32
134147	2	1	16	64	>512	256	128	64
136160	1	2	2	16	>512	256	128	256
137454	4	1	2	4	>512	32	32	16
137464	>4	64	8	>256	>512	512	128	256
138074	1	1	1	16	>512	128	128	64
138215	1	8	1	64	>512	128	128	128
181131	2	8	4	256	>512	256	64	32
181243	4	1	4	<4	16	<4	<4	<0,5

a The values are the means of three independent assays.

Antimicrobial agent abbreviations: IMI, Imipenem; CEF, Cefepime; TET, Tetracycline; CHL, Chloramphenicol; NAL, Nalidixic acid; NFL, Norfloxacin; OFL, Ofloxacin; SPA, Sparfloxacin.

**Table 3:** Percentages of *Enterobacter aerogenes* resistant strains.

Year	Number of strains	Number of resistant strains							
		IMI	CEF	TET	CHL	NAL	NFL	OFL	SPA
	MIC (µg/ml)	>8	>32	>8	>16	>16	>1	>1	>2
1995	45	2	6	8	29	39	37	34	36
2003	48	0	2	5	28	38	37	35	37
Total	93	2	8	13	57	77	74	69	72
%		2.1	8.6	13.9	61.3	82.7	79.5	74.1	78.4

Antimicrobial agent abbreviations: IMI, Imipenem; CEF, Cefepime; TET, Tetracycline; CHL, Chloramphenicol; NAL, Nalidixic Acid; NFL, Norfloxacin; OFL, Ofloxacin; SPA, Sparfloxacin.

### PAßN-susceptible efflux pump in *E. aerogenes*

We have previously demonstrated that the efflux pump inhibitor phenylalanine-arginine ß-naphthylamide (PAßN) is able to block the efflux involved in chloramphenicol resistance in *E. aerogenes* clinical strains [13], [24]. In order to evaluate the activity of this efflux mechanism in the two collections of *E. aerogenes* isolates, the effect of PAßN was assayed on the level of chloramphenicol susceptibility. For a number of isolates, addition of PAßN caused an increase in chloramphenicol susceptibility by at least three dilution factors (*e.g.* reduction of the MIC from 8 to 1 µg/ml) indicating the involvement of a PAßN-susceptible efflux in the resistance. In presence of PAßN, the MIC for chloramphenicol was noticeably decreased in about 40% of all non-susceptible (intermediate+resistant strains, I+R) isolates tested (Table 4). This result reflects the prevalence of a functional efflux mechanism that is susceptible to the inhibitor in the collected strains.

**Table 4:** Effect of EPI on chloramphenicol susceptibility of *E. aerogenes* strains.

Year	Number of strains	I+R ^a^	EP ^b^ susceptible I+R	EP insusceptible I+R
1995+2003	93	70	29	41
1995	45	32	9	23
2003	48	38	20	18

I, R ^a^: Intermediate, Resistant.

EP ^b^: Efflux Pump susceptible when PAßN induces a decrease of at least 3 dilutions.

It is important to note that among the total of I+R strains exhibiting a PAßN-susceptible efflux mechanism, the majority were isolated in 2003: the EPI-reversed strains were observed in a significantly greater number of chloramphenicol resistant strains (Table 4) selected in 2003 (20/29) than those selected in 1995 (9/29). These results indicate a noticeable increase in number of PAßN-susceptible efflux pumps acting in the strains isolated in 2003.

### Evidence of active efflux for chloramphenicol in multiresistant *E. aerogenes*

The activity of efflux pump in resistant isolates harbouring a PAßN-susceptible efflux was evaluated by measuring the intracellular accumulation of radiolabelled chloramphenicol in the reference strain and selected clinical isolates. Carbonyl cyanide *m*-chlorophenylhydrazone (CCCP), was a well-described tool allowing to characterize an energy-dependent efflux in resistant Gram-negative bacteria [16], [23], [24]. The variation of intracellular drug accumulation was evaluated in the absence and in the presence of this membrane energy uncoupler.

**Figure 2:**
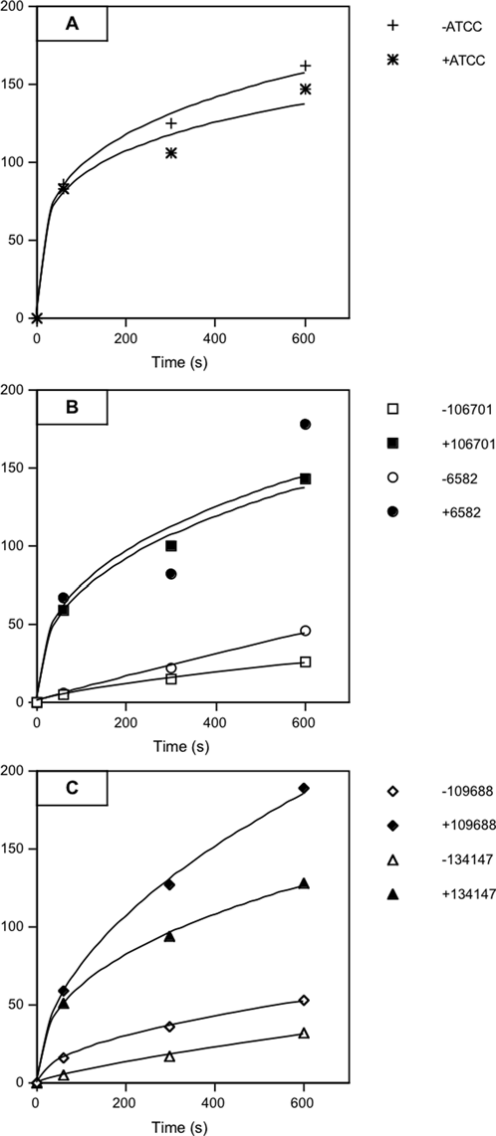
Uptake of ^14^C chloramphenicol by *E. aerogenes* strains. Accumulation of [^14^C] chloramphenicol was measured in ATCC type strain (A) and in clinical strains isolated during the years 1995 (B) and 2003 (C). ATCC13048 in the absence (+) and in the presence (*) of CCCP; strain 106701 in the absence (□) and in the presence (▪) of CCCP; strain 6582 in the absence (○) and in the presence (•) of CCCP; strain 109688 in the absence (◊) and in the presence (⧫) of CCCP; strain 134147 in the absence (▵) and in the presence of (▴) CCCP. Each point is the mean of three independent experiments. Values were plotted as cpm/OD_600_ over time (s).

Different strains representative of the two series were assayed in addition to the susceptible ATCC strain. By comparison to the ATCC strain, a 65% reduction in the chloramphenicol accumulation was detected in all tested chloramphenicol resistant *E. aerogenes* strains (Figure 2). This accumulation was significantly increased in the presence of CCCP during the incubation: 3.5 to 5 fold increase of intracellular chloramphenicol was obtained in the presence of the uncoupler (Figure 2). These results indicate that an active efflux pump is involved in chloramphenicol resistance in these isolates causing a decrease of intracellular drug concentration (Table 5).

**Table 5:** Antimicrobial susceptibilities and chloramphenicol accumulation in *E. aerogenes* strains.

Strains (year)	MIC (µg/mL)									Ratio of CHL accumulation a
	OFL	NFL	CIP	SPA	NAL	CHL	TET	CEF	IMI	
EA6582 (1995)	128 (32)	256 (256)	128 (64)	256 (32)	>512 (512)	256 (64)	4	4	4	3.9
EA106701 (1995)	128 (32)	256 (256)	64 (64)	512 (32)	>512 (256)	>256 (64)	8	2	2	5.0
EA109688 (2003)	64 (32)	128 (128)	64 (32)	64 (4)	>512 (256)	16 (1)	2	4	1	3.5
EA134147 (2003)	128 (16)	256 (256)	64 (32)	64 (4)	>512 (512)	64 (<4)	16	1	2	4.0
ATCC 15038 b	N.D c	0.12 (0.12)	N.D	0.06	N.D	2 (1)	0.5	0.25	0.25	1.1
EAEP294 b	N.D	64	16	N.D	1024 (128)	32 (32)	<0.25	N.D	N.D	N.D
EAEP289 b	N.D	256	32	N.D	1024 (256)	1024 (128)	8	N.D	N.D	N.D
EA27 b	>128	256 (128)	32	>64	>128	512 (64)	16 (2)	N.D	N.D	3.0

a Ratio of chloramphenicol accumulated at 600 s with and without CCCP.

b *E. aerogenes* control strains used: ATCC 15038 reference strain that expresses a normal level of the AcrAB efflux system; EAEP294 (*acrA^−^* deleted mutant)and EAEP289 that exhibits overexpression of the AcrAB efflux system.

c N.D: not determined.

Antimicrobial agent abbreviations: IMI, Imipenem; CEF, Cefepime; TET, Tetracycline; CHL, Chloramphenicol; NAL, Nalidixic Acid; NFL, Norfloxacin; OFL, Ofloxacin; SPA, Sparfloxacin; CIP, Ciprofloxacin. Values in parentheses were determined in the presence of efflux pump inhibitor PAßN at 26.3 µg/ml.

### Detection of an immunorelated-AcrA component of the efflux pump

To analyse the correlation between multidrug resistance and the presence of an efflux system we investigated the production of an immunorelated-AcrA component in the isolates exhibiting an energy dependent efflux of chloramphenicol [29], [30]. Regarding the expression of membrane transporters, we have recently demonstrated that a positive signal obtained from RT-PCR is not conclusive to conclude for the correct and functional production of a bacterial membrane protein [31]. It is important to mention that for membrane proteins (such as efflux transporters), several post-translational steps down-regulate their production in addition to the transcriptional regulation of their genes [31], [32]. There are the reasons for which the production of an active efflux immunorelated to the AcrAB-TolC system was checked by western blot analysis using an anti-AcrA polyclonal antibody as previously described [33]. We observed the presence of an immunorelated-AcrA protein in a large number of resistant *E. aerogenes* clinical isolates compared to *E. aerogenes* EAEP294 which contains an *acrA* gene knockout insertion. Interestingly, the isolate 54 presented no immunorelated-AcrA signal. Concerning EAEP294, it has been previously reported that this strain contains another efflux pump involved in antibiotic resistance and the anti-AcrA antibody is able to recognize an immunorelated-AcrA protein [33], [34]. Moreover, several clinical strains exhibited a high level of AcrA-like protein production similar to that observed in strain EAEP289, which has previously be shown to have an increased AcrA expression [21] and a high efflux pump activity (data not shown).

## Discussion

Emergence and evolution of drug resistance mechanisms in bacteria is an unavoidable phenomenon because it represents a natural adaptative response to environmental stress. Among the resistance mechanisms, high expression of drug efflux pumps is an underestimated mechanism which is contributing to resistance in clinical Gram-negative bacteria isolates of many pathogens such as *Pseudomonas aeruginosa* [35], [36], *Klebsiella pneumoniae* [29], [37] or *E. aerogenes* [13], [21].

The prevalence of an identified active efflux system in highly resistant clinical *E. aerogenes* strains collected, during 1995 and 2003, in a same area has been investigated. In a preliminary screen, the two collections exhibited a similar resistance profile for tetracycline, chloramphenicol, and the quinolones tested. In addition, molecular epidemiological typing indicates a relatively close relationship between the analyzed isolates and the previously major prevalent clone identified in different hospital infections due to *E. aerogenes* [4], [9], [11]. These results provide evidence for a stability in the resistance phenotype between 1995–2003 in the isolation site.

As previously reported, a broad spectrum efflux pump inhibitor, such as PAßN, may be used to discriminate between the resistance mechanisms involved in resistant isolates and to characterize the role of inhibitor-susceptible efflux pumps [22], [24], [38]. It has been demonstrated that PAßN may increase the activity of antibiotics which are expelled by efflux pumps such as AcrAB-TolC or MexAB-OprM [13], [22], [29], [38], [39]. This compound has been shown to restore the antibiotic intracellular concentration in several Gram-negative bacteria species including *E. aerogenes* [24], [30]. In all cases, the level of restoration of antibiotic susceptibility depends on the presence of additional resistance mechanisms such as target mutations or enzymatic barriers [30], [37], [39]–[41].

An active and PAßN-susceptible drug efflux system was evidenced in multiresistant *E. aerogenes* isolates. The activity of chloramphenicol efflux was determined in the presence and absence of PAßN, and the MICs were significantly decreased in the presence of PAßN in 40% of chloramphenicol resistant strains. In susceptible strains (MIC<8 µg/mL), the MICs were not noticeably affected by PAßN. These results indicate the presence of an efflux resistance mechanism that is reversed by PAßN addition in some chloramphenicol resistant strains. In these strains, the intracellular chloramphenicol concentration was influenced by CCCP, as the addition of this uncoupler of the proton motive force induced a substantial increase (3–5 fold) in the level of accumulated chloramphenicol. Moreover, in some isolates, a production of an immunorelated-AcrA efflux component was detected suggesting the involvement of a AcrAB-like efflux pump.

It is interesting to note that from the twenty nine strains that exhibit this PAßN-reversed efflux, twenty were isolated in 2003 and only nine were isolated in 1995. This variation indicated that the number of strains able to expel chloramphenicol had noticeably increased during this eight year interval. This is a major point taking into account that chloramphenicol is rarely used in clinical settings and the relative conservation of a resistant phenotype in the two tested collections. This result suggests that during this eight year period, the clinical environment, such as use of antimicrobial products and the consequent pressure on bacterial flora or colonizing bacteria, has selected strains expressing poly-selective efflux pumps since the FloR pump exhibits a specific activity for structurally-related phenicol drugs [42]. Several chemicals, *e.g.* biocides, bile salts or other efflux substrates are efficient agents for selecting bacteria that overexpress efflux pump mechanism [43]–[46]. This type of resistance survey is more restricted that global studies carried out in ESAC group [47], [48] or in the EARSS group [49] but yields interesting acute data concerning the evolution of one bacterial species and one resistance mechanism in a given hospital area. This selection allows us to a better approach of the antibiotic resistance evolution during the selected period. Regarding the resistance survey, it is important to mention that the COST Action BM0701(see http://www.cost.esf.org/bmbs) starting this year, is focused to the study of drug efflux mechanisms from clinical to chemical aspects.

After the emergence of extended spectrum ß-lactamases in *E. aerogenes* isolates and the recent characterisation of porin mutations in clinical isolates [2], [6], [11], [15], [50], this study describing an increase in inhibitor-susceptible efflux throws light on a new step in the evolution of mechanism in *E. aerogenes*. With the recent description of the key role of efflux pumps in favouring the acquisition of other resistance mechanisms in Gram-negative bacteria such as target mutation [25], [26], and in bacterial pathogenesis and fitness [51], [52], the increase of the number of clinical strains overproducing efflux pumps represents a serious emerging risk for the treatment of these infections.

## Methods

### Bacterial strains and growth media

A total of 93 strains of *Enterobacter aerogenes* were isolated in 1995 and 2003 in the hospitals of Marseille (France) from a variety of clinical specimens (respiratory tract, secretions, blood, urinary tract, etc …). They were selected according to their noticeable resistance against several ß-lactam antibiotics [10]. The *Enterobacter aerogenes* ATCC 15038 type strain was used as the reference (susceptible) strain for uptake of chloramphenicol. For immunodetection and characterization of efflux pumps, the strains EA ATCC 15038, EAEP289 (derivative of the clinical strain EA27) exhibiting norfloxacin efflux and the mutant EAEP294, which does not express AcrA were used [16], [21]. Bacteria were routinely grown in Luria Bertani (LB) or Mueller-Hinton (MH) broth at 37°C. The strain EA EP294 was grown in the presence of 50 µg/ml kanamycin.

### Antibiotic susceptibility tests

Susceptibility to imipenem, cefepime, nalidixic acid, norfloxacin, ofloxacin, ciprofloxacin, sparfloxacin, chloramphenicol and tetracycline was determined by the broth dilution method, as previously described [16] and according to the Clinical and Laboratory Standards Institute (http://www.clsi.org) and Comite de l'Antibiogramme de la SFM (http://www.sfm.asso.fr/nouv/general.phppa2). For the determination of MICs, approximately 10^6^ cells were inoculated into 1 ml MH broth containing twofold serial dilutions of each antibiotic. Results were read after 18 h at 37°C. The efflux pump inhibitor (EPI) phenylalanine-arginine ß-naphthylamide, PAßN [22], was used as previously described [24]. In this study, a fixed concentration of 26.3 µg/ml of EPI was selected for testing clinical isolates.

The MIC was the lowest concentration of the antimicrobial agent at which no growth was detected and resistance was defined as previously described [10], [13]. For chloramphenicol MIC≤8 corresponds to susceptible phenotype, 8<MIC<16 to an intermediate phenotype, and 16<MIC corresponds to resistant phenotype according to French Committee (http://www.sfm.asso.fr/nouv/general.phppa2). It is important to mention that, today no chloramphenicol breakpoint is proposed for *E. aerogenes* and *Enterobacter* spp by specific committee (European Antimicrobial Resistance Surveillance System, http://www.rivm.nl/earss/; European Committee on Antimicrobial Susceptibility Testing, http://www.srga.org/eucastwt/WT_EUCAST.htm). An efflux pump activity was identified when the PAßN addition induced a three fold decrease in MIC value for an antibiotic molecule [24], [29].

### Epidemiological typing

*E. aerogenes* typing was carried out by pulsed field gel electrophoresis (PFGE) with a CHEF-Mapper system (Bio-Rad France) as previously described [53]. Briefly, the *E. aerogenes* chromosomal DNA was digested with the restriction enzyme *Xba* I. Electrophoresis was performed for 22 hours with a pulsed time ranging from 1 to 50 s. Forty four clinical isolates from these two collections along with two clinical strains previously isolated, EA27 and EA3 [15], and the two EA ATCC strains 13048 and 15038 were studied for PFGE profile comparison.

### Measurement of chloramphenicol accumulation

Measurement of ^14^C chloramphenicol accumulation by intact cells has been described previously [13], [24]. Exponential-phase bacteria grown in LB broth were pelleted, washed once, and resuspended to a density of 10^10^ CFU/ml in 50 mM sodium phosphate buffer, pH7, containing 5 mM magnesium chloride. To de-energize the bacteria and block the efflux process, 50 µM carbonyl cyanide *m*-chlorophenylhydrazone (CCCP) was added 10 minutes before the radio-labelled chloramphenicol. Samples removed at set intervals were filtered and washed. The filters were dried and radioactivity was measured in a Packard scintillation counter.

### SDS-PAGE and immunodetection of AcrA

Exponential-phase bacteria in LB broth were pelleted and solubilized in boiling buffer at 96°C as described elsewhere [10], [13], [16]. Samples (amount corresponding to 0.02 optical density units at 600 nm) were loaded onto SDS-polyacrylamide gels (10% polyacrylamide, 0.1% SDS), then electrotransferred to nitrocellulose membranes [16]. Membranes were probed with antibodies raised against AcrA [33] (1∶50,000 dilution). Immunoreactive proteins were visualized with alkaline phosphatase -conjugated anti-rabbit secondary antibodies [13], [16].
